# Resistance status of *Anopheles gambiae* (*s.l.*) to four commonly used insecticides for malaria vector control in South-East Nigeria

**DOI:** 10.1186/s13071-020-04027-z

**Published:** 2020-03-24

**Authors:** Okechukwu Chukwuekezie, Emmanuel Nwosu, Udoka Nwangwu, Festus Dogunro, Cosmas Onwude, Nneka Agashi, Ebuka Ezihe, Clementina Anioke, Stephen Anokwu, Emelda Eloy, Peter Attah, Francis Orizu, Sylvester Ewo, Angela Okoronkwo, Anumba Joseph, Ijeoma Ikeakor, Sylvester Haruna, Virgile Gnanguenon

**Affiliations:** 1 National Arbovirus and Vectors Research Centre (NAVRC), Enugu, Nigeria; 2 Department of Biological Sciences, Kogi State University, Anyigba, Nigeria; 3 Centre de Recherche Entomologique de Cotonou, Cotonou, Benin

**Keywords:** Insecticide resistance, *Anopheles*, Knockdown resistance, Acetylcholinesterase-1 resistance, South-East Nigeria

## Abstract

**Background:**

Progress made in the control of malaria vectors globally is largely due to the use of insecticides. However, success in the fight against malaria has slowed down or even stalled due to a host of factors including insecticide resistance. The greatest burden of the disease is felt in Africa, particularly Nigeria. Unfortunately, adequate information on insecticide resistance is lacking in many parts of the country, particularly the South-East Zone. Hence, this study aims to bridge the information gap in the Zone.

**Methods:**

The study was conducted from April to December 2016. *Anopheles gambiae* (*s.l*.) larvae and pupae were collected from one community each, in the five states of the South-East Zone and reared to the adult stage. The adults were subjected to bioassays for insecticide resistance in accordance with the World Health Organization test procedures, across the four classes of insecticides used in public health. The mosquitoes were also subjected to molecular identification to the species level, and genotyped for West African knockdown resistance mutation (L1014F) and insensitive acetylcholinesterase-1 resistance mutation (G119S).

**Results:**

The mosquitoes were susceptible (100%) to bendiocarb but resistant to pirimiphos-methyl (39.6%), deltamethrin (57%) and dichlorodiphenyltrichloroethane (DDT) (13%). Molecular analysis revealed that only *An. gambiae* (*sensu stricto*) was found in all the states except for Ebonyi, where only *Anopheles coluzzii* was present. High frequencies (0.6–0.9) of the L1014F mutation were found across the zone. The L1014F mutation was significantly higher in *An. gambiae* (*s.s*.) than in *An. coluzzii* (*P* < 0.0001). A relatively low frequency (0.2) of the G119S mutation was found in *An. coluzzii*, and only in Ebonyi State.

**Conclusion:**

The results show that mosquitoes collected from the South-East Zone of Nigeria were resistant to all insecticides used, except for bendiocarb. The presence of L1014F and G119S resistance mutations reported in this study calls for urgent attention to stop the growing threat of insecticide resistance in the country.
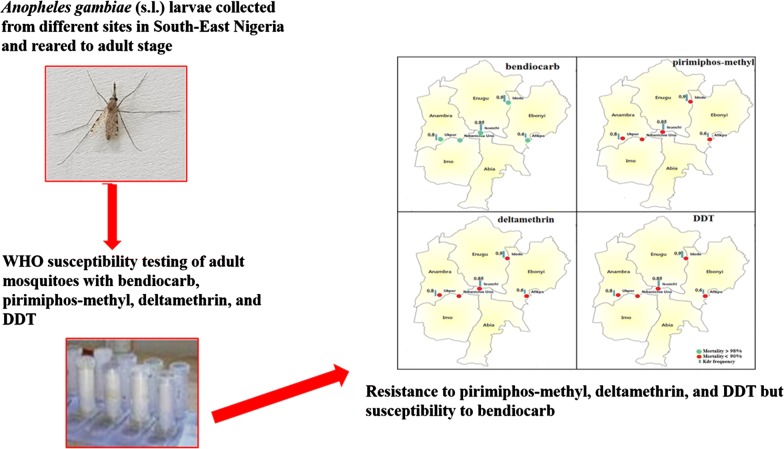

## Background

The fight against malaria yielded significant results between the year 2000 and 2015. Within this period, there was a global decline of 18% and 48% in malaria cases and deaths, respectively [1]. Beyond 2015, progress seems to have slowed and is almost stalled. According to the World Malaria Report 2018, there were 219 million cases and 435,000 deaths caused by the disease in 2017. In 2017, Africa accounted for an estimated 92% of global malaria cases and 93% of deaths [2]. The ten highest burden African countries recorded 3.5 million more cases in 2017 than they did in the year 2016 [2].

Nigeria alone accounted for about 25% and 19% of global totals of estimated malaria cases and deaths, respectively [2]. Of the 3.5 million more cases recorded by the highest burden countries, Nigeria accounted for 1.3 million (37%) cases. According to the WHO, there is an urgent need for accelerated reduction in incidence of the disease in high-burden countries, if global progress is to improve. Unfortunately, malaria incidence seems to be on the rise in Nigeria [2].

Vector control is a major pillar of malaria control. However, insecticide-treated nets (ITNs) and indoor residual spraying (IRS) are the mainstay of vector control, globally [3]. Until the year 2016, control of the malaria vectors hinges on the four classes of insecticides (pyrethroids, carbamates, organophosphates and organochlorines) approved by the World Health Organization Pesticide Evaluation Scheme (WHOPES). A new fifth class of insecticide (neonicotinoids) is now being used and tested in many countries and represent a new opportunity for vector control. The global decline in malaria cases and deaths observed between 2000 and 2015 is hugely attributed to use of insecticides in IRS and LLINs. Between 2013 and 2015, 93 million ITNs were distributed in Nigeria and around 46% of people were sleeping under ITNs (protected) in 2015 [4]. The IRS coverage was very low with only 2.5% all the states in the six geopolitical zones of Nigeria covered [5]. This information suggested an important gap of vector control tools that still need to be deployed based on local evidence on malaria vectors susceptibility to the insecticides.

The huge gains made in the fight against malaria, using these tools, is threatened by widespread insecticide resistance [1]. Between 2010 and 2017, the World Malaria Report 2018 shows that there was resistance to at least one insecticide in one malaria vector from at least one location in 68 of the 80 malaria endemic countries that reported standard monitoring data. According to the report, 22 (32%) of the countries detected resistance to all four classes, 16 (24%) to three classes, 19 (28%) to two classes and 11 (16%) to one class. Resistance to these four insecticide classes was detected in vectors present in all WHO regions except for the WHO European Region, although the extent of monitoring and prevalence of confirmed resistance to each insecticide class differed between regions. Incidentally, there is also widespread vector resistance to various classes of insecticides in Nigeria [6–11].

A relatively small amount of information is available on the vectors and their resistance status in southern Nigeria. The dearth of information on malaria vectors is worst in the southeast zone of Nigeria [12]. Some isolated studies in the South-East Zone have shown that the major malaria vector, *Anopheles gambiae* (*s.l*.), is resistant to three (organochlorine, pyrethroid and organophosphate) of the four classes of insecticides [9, 13]. However, these studies did not consider the various mechanisms driving insecticide resistance in the various locations.

This study sought to investigate the resistance status of malaria vectors and their underlying mechanisms, in the South-East Zone of Nigeria.

## Methods

### Study area

The South-East geopolitical zone is made up of 5 states (Abia, Anambra, Ebonyi, Enugu and Imo). It lies mainly in the forest belt of the country. There are two distinct seasons in this zone, the rainy season (April–November) and the dry season (December–March). The mean annual rainfall range is 3000–4000 mm, while the mean annual temperature range is 30.0–36.0 °C [12].

The 2006 national census estimates population of the zone to be 16,381,729. Population of the zone at present is estimated to be about 22,279,151 (assuming a national growth rate of 3%). With a total surface area of 29,095 km^2^, the South-East Zone has an estimated population density of 766 persons/km^2^.

**Fig. 1:**
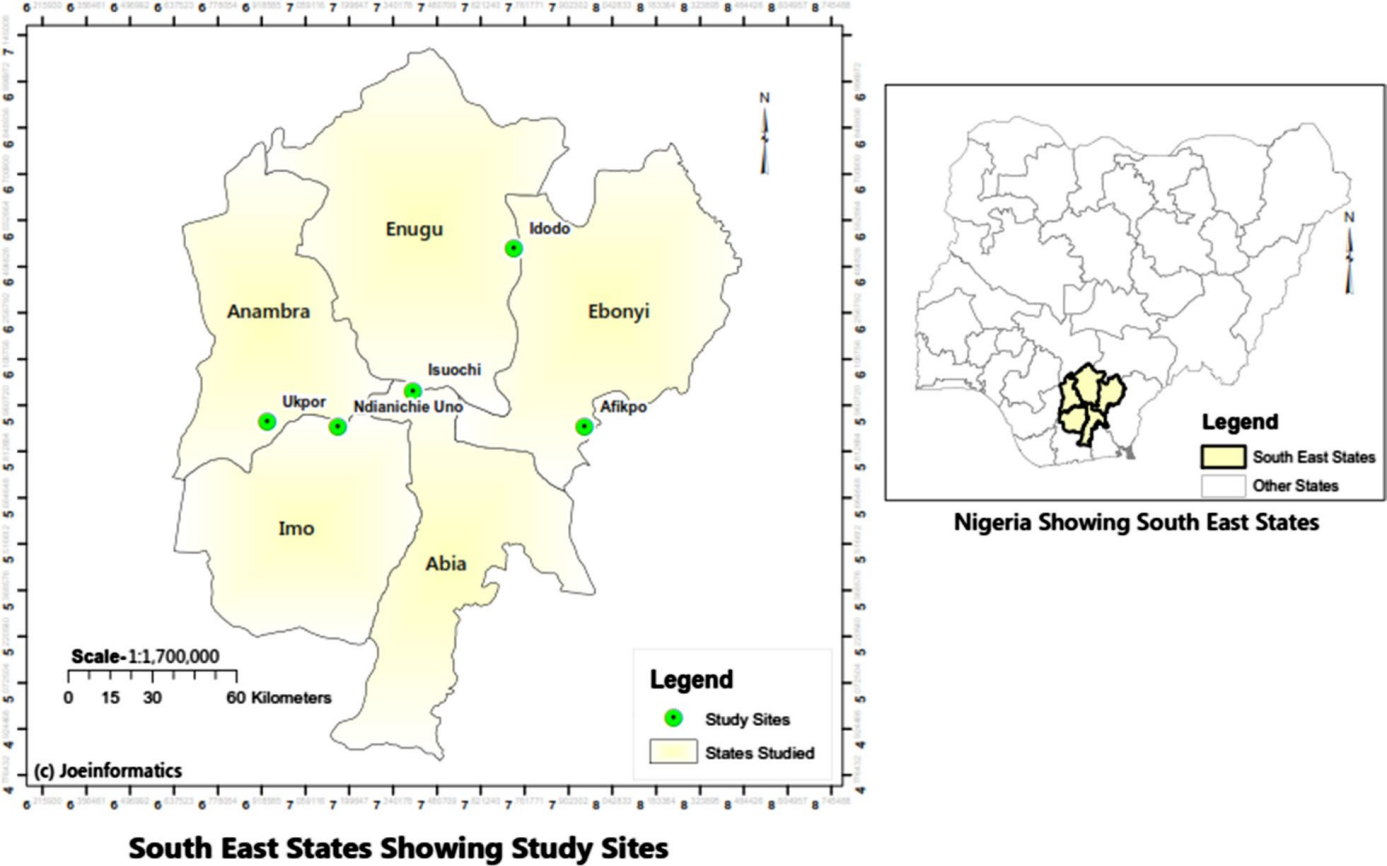
Southeast states showing study sites

The study was carried out in Isuochi, Umunneochi Local Government Area (LGA), Abia State (6.0057200° N, 7.4017600° E); Ukpor, Nnewi South LGA, Anambra State (5.9076300° N, 6.9343600° E); Afikpo, Afikpo North LGA, Ebonyi State (5.8895° N, 7.9538° E); Amaechi-Idodo, Nkanu East LGA, Enugu State (6.461253° N; 7.725586° E); and Ndianichie Uno, Ideato North LGA, Imo State (5.8897° N, 7.1629° E) (Fig. 1).

### Larval survey

The larvae collections were conducted from April (rainy season) to December (dry season) 2016. To ensure that populations of all possible vectors breeding in the study area were collected, extensive larval sampling was embarked upon. Puddles, rivers, streams, rice fields and other farms, containers, excavations, tire tracks, hoof prints and crab holes were sampled among others. Dippers, ladles, siphons and pipettes were used to ensure that each site in the study area was combed in the course of prospecting for larvae and pupae.

All larvae and pupae collected were placed in labeled containers according to the site of collection and transported to the insectary at the National Arbovirus and Vectors Research Centre (NAVRC), Enugu, Nigeria. They were then reared to adulthood, for susceptibility tests. The larvae were fed with fish feed (Tropical Flakes, Samyu Pets Corp, Shulin City, Taiwan) in 500 ml larval bowls. On emergence, the adult mosquitoes were fed with a 10% sugar solution in cotton wool. Throughout this period, the relative humidity and temperature were maintained at 80 ± 10% and 25 ± 2 °C, respectively.

### WHO susceptibility test

To determine the resistance status of the malaria vectors, non-blood-fed *An. gambiae* (*s.l*.) females that were 3 to 5 days post-emergence were used for susceptibility tests according to the WHO test procedures [14]. Adults of *An. gambiae* (*s.l*.) from all sites in the study area were tested against 0.25% pirimiphos-methyl (organophosphates), 0.1% bendiocarb (carbamates), 0.05% deltamethrin (pyrethroids) and 4% DDT (organochlorines). A total of 100 mosquitoes were exposed for 60 min to each insecticide (using four replicates) and 50 mosquitoes for the controls (using two replicates). The test conditions were maintained at a temperature of 26 ± 3 °C and a relative humidity of 74 ± 4%. Mosquitoes that survived were preserved for target site resistance (kdr-w and Ace-1^R^) assays, using silica gel in Eppendorf tubes.

### Morphological identification of vector species

All adult mosquitoes subjected to the test were identified at the NAVRC laboratory using the keys of Gillies & DeMeillon [15], Gillet [16] and Gillies & Coetzee [17].

### Molecular identification of *Anopheles* species

#### DNA extraction

Around 30 *An. gambiae* (*s.l*.) were selected randomly by site from live and dead mosquitoes (15 from pirimiphos-methyl tests and 15 from deltamethrin tests) and subjected to polymerase chain reaction (PCR) amplification for species identification and resistance mechanism detection. Genomic DNA from whole female mosquitoes was extracted according to the standard procedures of Collins et al. [18]. Extracted DNA was resuspended in 50 µl PCR grade water.

#### PCR for molecular species identification

PCR tests were performed with universal and species-specific primers for the *An. gambiae* complex. The SINE 200 PCR protocol [19] was used to simultaneously identify members of the *An. gambiae* complex and their former molecular forms.

#### PCR for resistance mechanisms detection

PCR analyses were performed at the Center of Entomological Research of Cotonou, the Republic of Benin. The PCR-restriction fragment length polymorphism diagnostic test was performed to identify the presence of the L1014F mutation (*kdr*) using the method described by Martinez-Torres et al. [20]. The following primers were used: Agd1 (5′-ATA GAT TCC CCG ACC ATG-3′), Agd2 (5′-AGA CAA GGA TGA TGA ACC-3′), Agd3 (5′-AAT TTG CAT TAC TTA CGA CA-3′) and Agd4 (5′-CTG TAG TGA TAG GAA ATT TA-3′). The PCR was performed in a 25 μl mixture including 17.1 μl distilled water, 2.5 μl 10× PCR buffer (TEKNOVA, California, USA), 0.3 μl MgCl_2_, 1 μl of each primer, 1 μl of dNTP and 0.125 μl of kappa Taq DNA polymerase (Kapa Biosystems, Cape Town, South Africa). The cycling conditions were initially set as 95 °C denaturation for 3 min, followed by 10 cycles of 1 min at 94 °C, 30 s at 54 °C and 30 s at 72 °C. This was followed by 30 cycles of 1 min at 94 °C, 30 s at 47 °C and 30 s at 72 °C, with a final extension step at 72 °C for 10 min. The PCR products were verified on a 2% agarose gel and stained with ethidium bromide for visualization using a Syngene bio-imaging system (Syngene, Cambridge, UK). G119S mutation (Ace-1R) was detected using the method described by Weill et al. [21]. The primers MOUSTDIR1 (25 pmol/μl) (5′-CCG GGN GCS ACY ATG TGG AA-3′); and MOUSTREV1 (25 pmol/μl) (5′-ACG ATM ACG TTC TCY TCC GA-3′) were used. The G119S mutation PCR was performed in a 25 μl mixture including 16.35 μl sterile water, 5.0 μl of 5× GoTaq PCR Buffer (Promega, Wisconsin, USA), 1.0 μl dNTP, 1.25 μl of each primer and 0.15 μl Taq DNA. The cycling conditions were 93 °C/5 min × 1 cycle, (93 °C/1 min -0–53 °C/1 min -0–72 °C/1.5 min) × 35 cycles, 72 °C/10 min × 1 cycle, and 4 °C of holding temperature. A total of 15 µl of the PCR product was digested with 1 μl *Alu*I restriction enzyme, 2 μl of H_2_0, and 2 μl buffer. The incubation period was 37 °C for 8–24 h. Following incubation, 5 µl of the digest was analysed on 2% agarose gel and stained in ethidium bromide.

### Data analysis and interpretation

The resistance status of mosquito samples was determined according to the WHO protocol for insecticide resistance monitoring [14] as follows: mortality rate > 98%, the population was considered fully susceptible; mortality rates of 90–98%, resistance suspected in the population; mortality rates < 90%, the population was considered resistant to the tested insecticides

Abbott’s formula should be used to correct the observed mortality when the mortality in the control is between 5–20% [22]. However, the mortality rates in all controls were less than 5% during the tests and the use of Abbott’s formula was not required to correct mortality rates.

The Fisher’s exact test was used to compare mortality rates among the mosquito populations using GENEPOP software [23]. To assess variability of the allelic frequencies of the *kdr* (L1014F) mutation and Ace-1^R^ (G119S) across populations, the genotypic differentiation test was performed [24].

## Results

### Susceptibility of *Anopheles gambiae* (*s.l*.) from the South-East Zone to deltamethrin (pyrethroid) and DDT insecticides

**Fig. 2:**
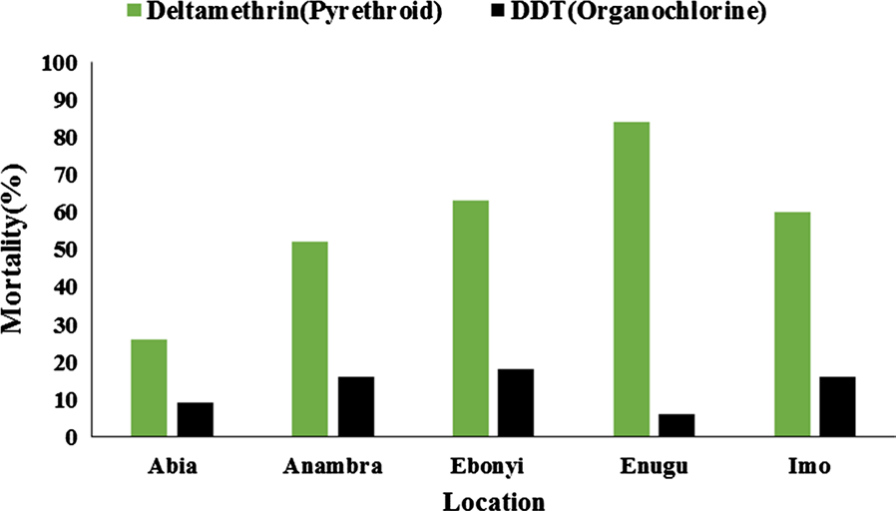
Observed mortalities (with 95% confidence interval) of *An. gambiae* (*s.l*.) with deltamethrin and DDT across the states

**Fig. 3:**
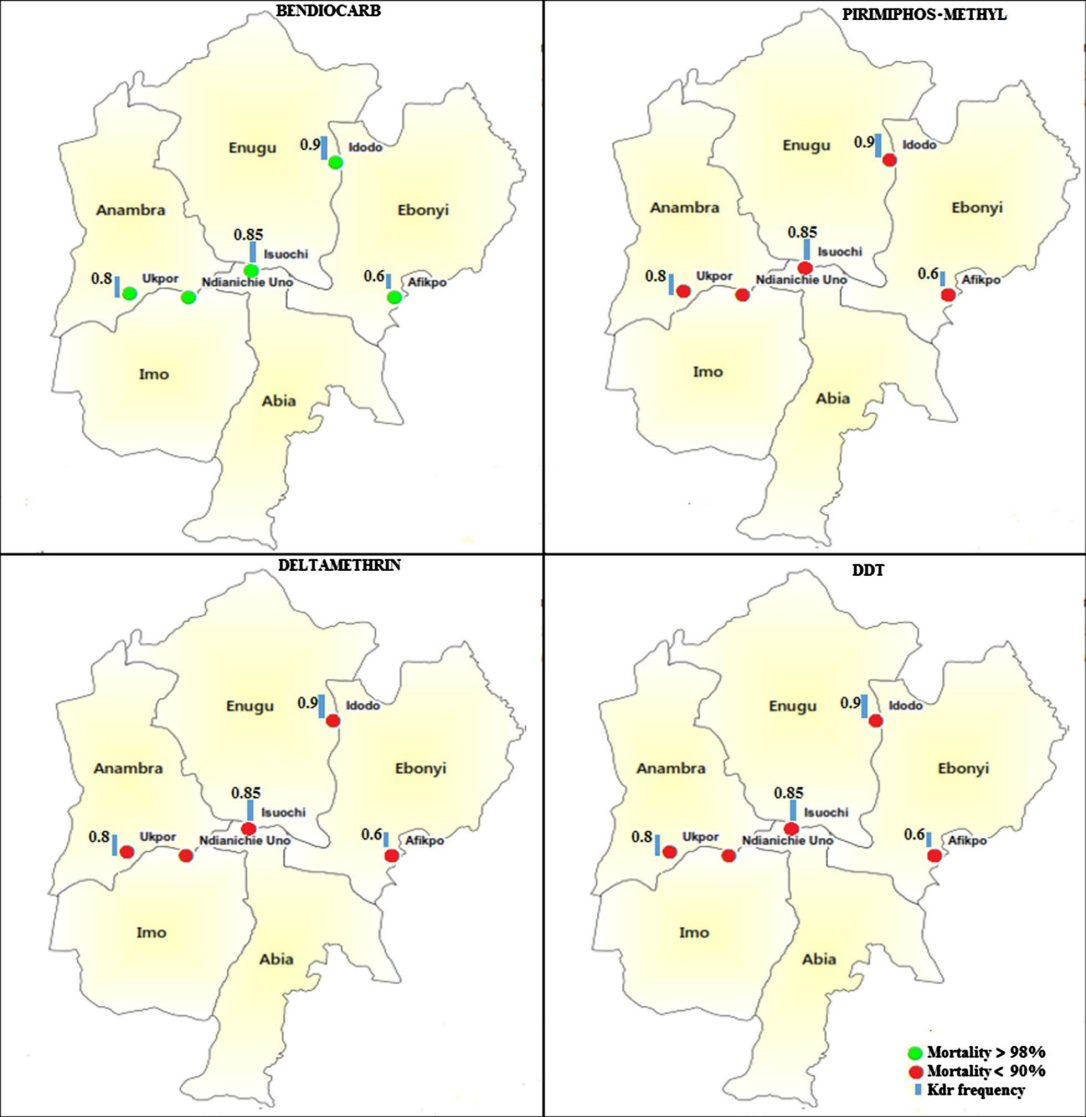
Distribution of insecticide resistance in South-East Nigeria

The average mortality of *An. gambiae* (*s.l*.) populations tested with DDT was 13% (95% CI: 6.55–19.45%). Observed mortality in the states was as follows: Enugu State: 6% (95% CI: 2.23–12.60%); Abia State: 9% (95% CI: 4.19–16.39%) (Fig. 2); Anambra and Imo States: 16% (95% CI: 9.43–24.67%). The highest mortality with DDT was observed in Ebonyi State (18%; 95% CI: 11.03–26.94%) (Figs. 2, 3).

For pyrethroid and deltamethrin, the average mortality observed was 57% (95% CI: 30.95–83.05). This mortality varied from one state to another. The observed mortality with deltamethrin was 26% (95% CI: 17.74–35.73%) in Abia State (Fig. 2), 52% (95% CI: 41.77–62.09%) in Anambra State, 60% (95% CI: 49.72–69.67%) in Imo State and 63% (95% CI: 52.76–72.44%) in Ebonyi State. The highest mortality with deltamethrin was 83% (95% CI: 75.32–90.56%) and was observed in Enugu State (Figs. 2, 3).

### Susceptibility of *Anopheles gambiae* (*s.l*.) from the South-East Zone to bendiocarb (carbamate) and pirimiphos-methyl (organophosphate) insecticides

**Fig. 4:**
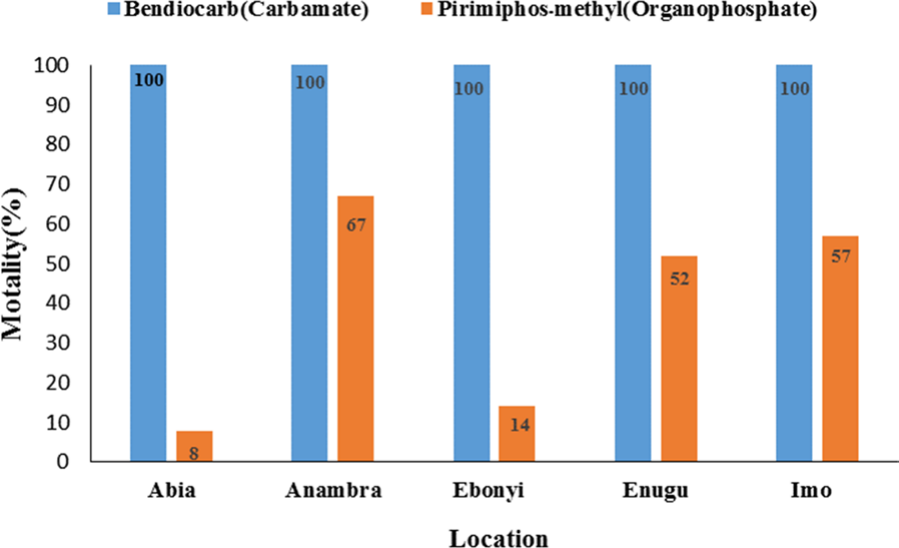
Observed mortalities (with 95% confidence interval) of *An. gambiae* (*s.l*.) with pirimiphos-methyl and bendiocarb across the states

The average mortality observed with bendioacarb was 100%. A mortality rate of 100% was observed in all states assessed (Figs. 3, 4).

With the organophosphate pirimiphos-methyl, the average mortality observed across all states was 39.6% (95% CI: 6.4–72.8%). At a state level, the observed mortalities were 8% (95% CI: 3.51–15.15%) in Abia State, 14% (95% CI: 07.87–22.37%) in Ebonyi State, 52% (95% CI: 41.77–62.09%) in Enugu State and 57% (95% CI: 46.71–66.86%) in Imo State (Fig. 4). The highest mortality with pirimiphos-methyl was observed in Anambra State with a mortality of 67% (95% CI: 56.88–76.08%) (Figs. 3, 4).

### Distribution of sibling species of *Anopheles gambiae* (*s.l*.) across the states

All *An. gambiae* (*s.l*.) tested from Abia, Anambra, Ebonyi and Imo States were identified to be *An. gambiae* (*s.s*.) (Table 1). On the contrary, the mosquitoes tested from Ebonyi State were all *Anopheles coluzzii* (Table 1). Samples from Enugu were not subjected to testing in this assay.

**Table 1:** Distribution of *An. gambiae* (*s.s*.) and *An. coluzzii* across the states

Species	Abia	Anambra	Ebonyi	Imo
*An. gambiae* (*s.s.*) (%)	100	100	0	100
*An. coluzzii* (%)	0	0	100	0
Total	30	30	30	30

### Distribution of L1014F and G119S mutations in *kdr* genes across the states

Target site resistance was assayed in the mosquitoes collected from four of the five South-East states. The GII9S mutation was detected at low frequencies (0.2) in mosquitoes collected from Ebonyi State only (Table 2). This mutation was detected in its heterozygote state in *An. coluzzii* which seemed to be breeding in allopatry in the state. On the other hand, mutations in the *kdr* gene were detected in *Anopheles* mosquitoes collected from all states tested (Table 3). The resistance frequencies were high in both *An. coluzzii* which recorded a frequency of 0.6, and *An. gambiae* (*s.s*.) which had an average frequency of 0.85 across all states from which they were collected (Table 4). The frequency of *kdr* resistance in *An. gambiae* (*s.s*.) was significantly higher than that of *An. coluzzii* (*P* < 0.001) (Table 4).

**Table 2:** Frequency of *Ace-1* resistance across the states

Location	*n*	RR	RS	SS	F (Ace-1^R^)
Afikpo, Ebonyi State	30	0	6	24	0.2
Isuochi, Abia State	30	0	0	30	0
Ndianiche-Uno, Imo State	30	0	0	30	0
Ukpo, Anambra State	30	0	0	30	0
Total	120	0	6	114	0.025

*Abbreviations*: n, number tested; SS, homozygous susceptible; RS, hybrid resistant and susceptible; RR, homozygous resistant; F, frequency

**Table 3:** Frequency of knock-down resistance mutation (*kdr*) across the states

Location	*n*	RR	RS	SS	F (*kdr*)	Odds ratio	95% CI	*P*-value
Afikpo, Ebonyi State	30	12	12	6	0.60	–	–	–
Isuochi, Abia State	30	24	3	3	0.85	3.70	1.57–9.39	0.002
Ndianiche-Uno, Imo State	30	24	6	0	0.90	5.81	2.26–17.26	< 0.001
Ukpo, Anambra State	30	18	12	0	0.80	2.63	1.17–6.16	0.018
Total	120	78	33	9	0.79			

*Abbreviations*: SS, homozygous susceptible; RS, hybrid resistant and susceptible; RR, homozygous resistant; F, frequency; CI, confidence interval

**Table 4:** Frequency knock-down resistance mutation (*kdr*) across mosquito species

Species	*n*	RR	RS	SS	F(*kdr*)	Odds ratio	95% CI	*P*-value
*An. gambiae* (*s.s.*)	90	66	21	3	0.85	0.27	0.14–0.52	< 0.001
*An. coluzzii*	30	12	12	6	0.60			
Total	120	78	33	9	0.79			

*Abbreviations*: SS, homozygous susceptible; RS, hybrid resistant and susceptible; RR, homozygous resistant: F, frequency; CI, confidence interval

## Discussion

The present study is in part an update on the 2014 and 2015 insecticide resistance monitoring of the *An. gambiae* (*s.l*.) in Enugu State and an expansion to other states in the Zone. The expansion to other states is to bridge the enormous information gap on malaria vectors and their resistance status in the study area. The results revealed that *An. gambiae* (*s.l*.) populations were resistant to organophosphates, pyrethroids and DDT. They were only susceptible to the carbamates, particularly, bendiocarb. High knockdown resistance frequencies were observed in the mosquito populations of the study area. The acetylcholinesterase-1 resistance frequencies were low among the mosquito populations tested.

In 2014 and 2015, malaria vector populations showed the greatest resistance against DDT and then pyrethroids [9, 25]. This may be due to excessive use of DDT in the past, which may also have conferred resistance on the pyrethroids through cross-resistance. Additionally, LLINs have been widely distributed in the South-East Zone for about a decade. The fact that the pyrethroids are the only class of insecticide used in LLINs is likely to have exacerbated the situation. Across the five states, deltamethrin performed better than the organochlorine and organophosphate used in this study. This may not be reflective of the overall performance of the different insecticides in the pyrethroid class. Deltamethrin being a type II pyrethroid contains an alpha-cyano group allowing it to exert a better killing effect on insects [26]. However, results of this study corroborate with the recent finding in Amansea, Anambra State, by Nwankwo et al. [10], where malaria vectors were only susceptible to the carbamate, bendiocarb.

Widespread resistance of *An. gambiae* (*s.l*.) to DDT and pyrethroids has been reported by various studies in Nigeria [6–8, 11] and other parts of Africa [27–30]. This may be largely due to cross-resistance, use of agricultural insecticides and/or use of pyrethroids in LLINs and IRS. On the contrary, resistance to pirimiphos-methyl (organophosphate) is not as widespread as those of DDT and the pyrethroids. *Anopheles gambiae* (*s.l*.) susceptibility to the insecticide has been reported in some parts of South-West Nigeria [9, 10]. The vectors were found to be susceptible to both the organophosphates and carbamates in Lagos and Oyo States. However, resistance to pirimiphos-methyl has been recorded in several other geopolitical zones of Nigeria [9]. The high resistance observed with pirimiphos-methyl could be due to the fact that this insecticide was among the pesticides widely used in the previous years for crop control in agriculture as reported by Odeyemi et al. [31] and the West Africa Productivity Programme [32] and could have exerted a high selection pressure on mosquito larvae.

Target site resistance was the only mechanism of resistance analysed in this study. Nevertheless, target site resistance is a major mechanism driving vector insecticide resistance in Nigeria [33]. The West African knockdown resistance (L1014F) was found in high frequencies (between 0.6–0.9) across the states. The homozygous resistance state (RR) dominated the allelic frequency in all the states. *Anopheles gambiae* (*s.s*.) and *An. coluzzii* showed similar L1014F frequencies. This agrees with the findings of Gnanguenon et al. [29] and Okorie et al. [9], where the L1014F mutation was found in both populations. However, it contrasts a report by AIRS Nigeria [11] where populations of *An. coluzzii* collected from the study were all negative for the L1014F mutation.

In contrast to L1014F, acetylcholinesterase resistance (G119S mutation) was observed at a low frequency (0.2). It was only found in its heterozygous genotype (RS) state in an *An. coluzzii* population collected from Ebonyi State. The dominant *An. gambiae* (*s.s*.) tested from all the states were negative for G119S mutation. This may be the first time the insensitive acetylcholinesterase-1 mutation is detected in malaria vectors in Nigeria, as there has been no record of it between 2010 and 2016 [34] in the country. Our finding is in contrast with a study by Gnanguenon et al. [29] in Benin, where all *An. coluzzii* were negative to the insensitive acetylcholinesterase-1 gene. However, despite the very low frequency of the G119S mutation, organophosphate resistance was observed in all the states. This suggests that other resistance mechanisms such as metabolic resistance which confers cross-resistance to organophosphates and carbamates could be involved in the vector populations. This corroborates the studies by Djouaka et al. [34] and Awolola et al. [8] reporting multiple resistance mechanisms in malaria vectors in various locations of Nigeria. If the frequency of the G119S mutation is low (as identified in the present study) and esterase activity is high, it is possible for the vectors to be susceptible to the carbamates and resistant to the organophosphates [35]. This could occur because esterase activity exerts relatively little effect on the carbamates but a huge effect on the organophosphates. The presence of the G119S mutation needs to be significant alongside some other metabolic resistance for a possible expression of phenotypic resistance to carbamates. Hence, our findings suggest that esterase activity in the *Anopheles* populations tested may be high.

Our findings suggest that the vectors breed in allopatry in all the states tested. However, more studies should be carried out in all states, to better understand the spread of *An. gambiae* (*s.s*.) and *An. coluzzii*. Meanwhile, to the best of our knowledge, this is the first-time allopatric breeding for the two sibling species has been reported in such a large scale coordinated study in Nigeria. This is in contrast with the findings of Awolola et al. [7, 27], Okorie et al. [9] and Djouaka et al. [34] recording sympatric breeding of the two members of the *An. gambiae* complex.

Despite of the significant findings observed in the study, it does have its limitations. The intensity of the observed resistance was not assessed. The presence of L1014S and N1575Y mutations which are important target site mutations in *An. gambiae* (*s.l*.) was not documented. The metabolic resistance and transcriptional analyses for metabolic resistance mechanisms were not carried out in the study and constituted an important limitation. The inclusions of these resistance mechanisms would have provided a clear profile of insecticide resistance in southeast Nigeria. However, a recent study conducted by Ibrahim et al. [36] demonstrated that metabolic resistance (CYP450) is present in the malaria vector population with a synergist significant recovery observed after exposure of wild *An. gambiae* (*s.l*.) from the Sahelo-Sudanian region of northern Nigeria to synergist plus deltamethrin. Biochemical analyses in another study from surveillance sites of Nigeria [37] demonstrated a significant increase in the levels of P450 enzymes in a resistant *An. gambiae* population from Lagos, Ogun and Niger states. The same study also showed the presence of glutathione S-transferase (GST) mechanism in Lagos and Ogun revealing an increasing presence of metabolic resistance (P450 + GST) in malaria vectors from Nigeria. These findings are also confirmed by Fagbohoun et al. [38] who demonstrated that cytochrome P450 mono-oxygenase resistance was highly involved in the resistance of *An. gambiae* to DDT and pyrethroids.

The presence of the L1014S-kdr mutation has been recently reported in a single *An. arabiensis* from the Sudan savannah of northern Nigeria by Ibrahim et al. [39]. However, Habibu et al. [40] have reported a higher frequency of L1014S-kdr mutation in *An. coluzzii* and *An. arabiensis* mosquitoes than previously reported by Ibrahim et al. [39]. These findings suggest an increasing L1014S-kdr mutation rate in Nigeria. As yet, the N1575Y mutation has not been reported in Nigeria but has been reported in the West African regions of Côte d’Ivoire and Burkina Faso and could therefore potentially spread to Nigeria [41, 42]. In the present study, only a small number of samples (*n* = 30) were used for PCR due to limited funding to perform the analysis and this represented an important limitation. A more accurate estimation of resistance mechanisms could have been obtained using a larger sample size.

Monitoring insecticide resistance is an important strategy against malaria vectors and the resistance management strategies depends on the evidences of local vector resistance to insecticides. This data will be beneficial to the National Malaria Control Programme, as the results will assist them with their choice of insecticide for use in southeast Nigeria. A pro-active strategy should be used to avoid vector resistance to bendiocarb. Alternative new insecticides such as neonicotinoids (clothianidin and a combination of clothianidin/deltamethrin) could be used in rotation or mosaic with bendiocarb as a local resistance management strategy.

## Conclusions

The study demonstrated a widespread resistance to three of the four classes of insecticides used in public health in the South-East Zone of Nigeria. This could have huge implications in the control of malaria and its vectors in the Zone, as most of the vector control interventions rely heavily on insecticides from these classes. There is an urgent need for implementation of insecticide resistance management strategies in the Zone to assess the spread of resistance. Moreover, the detection of G119S resistance supports the urgent need of an insecticide resistance management plan. The spread of G119S resistance will expand malaria vector resistance to the remaining class of insecticide (carbamate). Hence, there is a need for continuous and expanded insecticide resistance monitoring in the Zone to obtain a broader and clearer idea of the situation.

## Data Availability

All data generated or analyzed during this study are included in this published article.

## Abbreviations

LGA: Local Government Area
NAVRC: National Arbovirus and Vectors Research Centre
*s.l.*: *sensu lato*
